# A Funny Symptom—Sulphur 55^m^

**Published:** 1890-11

**Authors:** M. A. A. Wolff

**Affiliations:** Gainesville, Tex.


### A FUNNY SYMPTOM—SULPHUR 55$^{M}$.

M. A. A. Wolff, M. D., Gainesville, Tex.

(Continued from September No., page 407.)

General symptoms, additional to such stated in first report: Pain all the time in perineum from dragging of scrotum at the incision-scar, and inside when clogged up, in which case there is also pain in left renal region. The urine does not dribble in the rag wrapped around penis, but trickles out as from a spring; but when standing up and trying to have it come in a stream it will only dribble, except when after one or several hours' sleep, *during which time there is no discharge of urine*, it will escape in a stream; sometimes too, not always, as it used to be, some urine will be discharged from anus. (Rags were instituted instead of urinal, which blistered and excoriated glans.)

Special Symptoms. August 5th.—Discharge of much pus; urination very free (in the rags) all day. Formerly urination prevailed during night, now as stated. A stool not hard but regularly shaped, breaks in two by itself, and at place of breakage shows a piece of something looking like decayed flesh from a wound after amputation. Heavy erections awoke me three times during the night. Got up and abundant urine through urethra.

August 7th.—Dropsy of legs goes gradually down.

August 13th, five A. M.—Immense diarrhœa and discharge of fæces through urethra. From seven A. M. no urination, something seems to clog the urethra; pressure brings out pus like a gonorrhœal discharge.

August 15th, two A. M., four A. M., and six A. M.—Immense diarrhœa, grayish, mushy, passes partly through urethra.

August 18th.—After having been constipated (without feeling of urging) since the 15th A. M., a normal, very large stool, sound in every respect and discharged without pain. But for some heaviness in perineum no more trouble from pile, or, as I contest, prolapsus recti.

August 19th.—Twice a good stool, but first time preceded by discharge of a brownish, jelly-like mucus, some of which escapes through urethra. Feeling as if there were more to come. At eleven A. M., temperature one hundred degrees, was normal at six A. M.; at twelve noon one hundred and one. Reported in writing to Professor Reed. From nine P. M. to twelve a decided Baptisia fever, but would not interfere with the Sulphur. After twelve, when I had been up urinating, perspiration. The height of the fever, one hundred and two degrees, however, remained. Old symptoms returned, as the spluttering from the mouth, gushing of urine from anus, short cough, painful to pubic region (root of urethra), only once causing a few drops of urine to dribble. No discharge of urine now when lying down, either during sleep or *being awake* (new symptom).

August 20th, eleven A. M.—Received the third dose of Sulphur (since July 18th), viz.: CM. I kept the bed all day and following night, only getting up to urinate. During the day, after taking the Sulph., temperature rose to one hundred and three degrees. At night it had fallen to one hundred and one and a half.

August 20th.—Temperature ninety-nine and two-fifths. Urination only through urethra. *Dropsy seems completely gone.* Stayed up until noon. Through over-exertion, I think, temperature rose to one hundred and two during the day. Another cause for it may be an abscess which, I supposed from my sensations, in the part bound by the recto-vesical fistulæ. Sitting down that forenoon did not, as hitherto, cause involuntary urination. At dinner-time took a bowl of soup and then went to bed. Up once that afternoon, discharging urine in a thin stream. Half-past five P. M. got up in the chair again, when, as soon as I sat down, the tickling re-commenced. Temperature one hundred and one and three-fifths. No stool since 19th (another possible cause of fever). Six P. M., felt urging to stool. Digital examination had already yesterday proven that there was a hard stool lodged in the bowels. Now, urine must have been accumulating from the lesion of the bladder behind this stool and have dissolved some of it, for, when I sat down on the commode, there was a gushing from anus of urine colored by fæces, which also escaped through urethra. It burned like fire. At last there came a hard, sound stool.

August 22d, A. M.—Have slept well all night; got up three times to urinate, the first two gushing from anus, the third only from urethra. Put on this morning, for the first time, socks and slippers with greatest ease. Good normal stool at 8.30 A. M. The most unpleasant feeling is caused by excoriation of glans around meatus. No appetite. At bedtime temperature one hundred and one degrees.

August 23d.—Had a good night; fever gone.

August 26th.—The wet rags caused the excoriation of glans. Small compresses dipped in Calendula water, in spite of being wet with urine, healed it in two days. So I thought to employ them in the urinal. It works like a charm.

August 27th.—Feel very well, after a good sleep during the night.

August 29th.—Again some aggravation; temperature one hundred and one degrees.

August 30th.—Same as yesterday, but more aggravation.

September 1st.—Received fourth dose of Sulphur CM. It did not take effect before the 3d.

September 5th.—Left hospital. From 5th to 10th only harassing trouble is excoriation of the urethra and glans, as well as below the same.

September 10th, P. M.—Took the cars for home. Arrived Friday 4.50 A. M. Only trouble the excoriation (and the involuntary urination).

September 14th.—Felt bad; temperature one hundred and two. Fifth dose Sulph. CM acted well in less than half an hour.

To-day, September 17th, would be all right if it were not for the involuntary urination and excoriations. The fistula is decidedly improving.

---

SULPHUR.—Excoriations of genitals and inside of thighs. The flow of urine painful to parts passed over.—*Guernsey's Key-note.*

